# Water-Soluble Lead Sulfide Nanoparticles: Direct Synthesis and Ligand Exchange Routes

**DOI:** 10.3390/nano14141235

**Published:** 2024-07-22

**Authors:** Saar Pfeffer, Vladimir Ezersky, Sofiya Kolusheva, Yuval Golan

**Affiliations:** 1 Department of Materials Engineering, Ben-Gurion University of the Negev, Beer-Sheva 8410501, Israel; saarpf@post.bgu.ac.il; 2 Ilse Katz Institute for Nanoscale Science and Technology, Ben-Gurion University of the Negev, Beer-Sheva 8410501, Israel; ezersky@bgu.ac.il (V.E.); kolushev@bgu.ac.il (S.K.)

**Keywords:** ligand exchange, nanoparticles, polyvinylpyrrolidone, lead sulfide

## Abstract

Colloidal semiconductor nanoparticles (NPs) represent an emergent state of matter with unique properties, bridging bulk materials and molecular structures. Their distinct physical attributes, such as bandgap and photoluminescence, are intricately tied to their size and morphology. Ligand passivation plays a crucial role in shaping NPs and determining their physical properties. Ligand exchange (LE) offers a versatile approach to tailoring NP properties, often guided by Pearson’s Hard–Soft Acid–Base theory. Lead sulfide (PbS), a semiconductor of considerable interest, exhibits size-dependent tunable bandgaps from the infrared to the visible range. Here, we present two methods for synthesizing water-soluble, polyvinylpyrrolidone (PVP)-coated PbS NPs. The first involves direct synthesis in an aqueous solution while utilizing PVP as the surfactant for the formation of nano-cubes with a crystal coherence length of ~30 nm, while the second involves LE from octadecylamine-coated PbS truncated nano-cubes to PVP-coated PbS NPs with a crystal coherence length of ~15 nm. Multiple characterization techniques, including X-ray diffraction, transmission electron microscopy, Fourier-transform infrared spectroscopy, and thermal gravimetric analysis, confirmed the results of the synthesis and allowed us to monitor the ligand exchange process. Our findings demonstrate efficient and environmentally friendly approaches for synthesizing PVP-coated PbS NPs.

## 1. Introduction

Colloidal semiconductor nanoparticles (NPs) are commonly characterized by an inorganic core comprising a range of several hundred to a few thousand atoms, encapsulated by an exterior organic layer of surfactant molecules, referred to as ligands [1]. These NPs exhibit attributes that position them as an emergent state of matter, bridging the gap between bulk materials and molecular structures. This unique classification stems from the fact that NPs, unlike bulk materials, exhibit distinctive physical properties, such as bandgap and photoluminescence, which are contingent upon their dimensions [2]. Due to the diminutive scale of colloidal NPs, the ratio between their surface area and volume becomes notably large. Consequently, the properties of NPs are profoundly governed by surface energy and surface states. Moreover, any occurrences of surface reconstruction and unsaturated dangling bonds can instigate the emergence of novel electronic states [3]. To mitigate the influence of external surroundings on NP properties, effective surface passivation of NPs is imperative. An approach commonly employed for this purpose involves the utilization of ligands. These ligands play a dual role: not only do they passivate the NP surface by saturating dangling bonds, but they also play a pivotal role in shaping the NP size, morphology, and crystal structure [4,5]. Ligands can manifest as organic molecules, polymers, or inorganic entities. Furthermore, their hydrophilic/hydrophobic nature dictates the stability of NPs in aqueous solutions or nonpolar solvents. The versatility of ligand passivation is underscored by the potential for post-synthesis ligand exchange (LE). This procedure entails the replacement of organic molecules on the NP surface with new ligands, thereby affording a means to tailor NP properties for specific applications [6]. For instance, a strategy involves initially synthesizing NPs with a morphology achievable solely through the utilization of hydrophobic ligands, followed by an LE process to substitute with hydrophilic ligands. This results in NPs retaining their unique morphology while becoming stable in aqueous environments [7]. A significant proportion of LE reactions adhere to Pearson’s Hard–Soft Acid–Base (HSAB) theory [8]. This theory categorizes Lewis acids and bases into “hard” and “soft” types. According to this theory, soft acids tend to interact with soft bases, giving rise to covalent complexes, whereas hard acids appear to associate with hard bases, yielding ionic complexes. In the context of a cation-rich lead sulfide NP surface, such as (111)A, lead cations, functioning as soft acids, preferentially interact with soft bases such as carboxylates or thiolates [9]. Consequently, for the exchange of ligands on the Pb^+^-terminated surface, incoming ligands must possess greater softness than the outgoing ones. An additional determinant of the ligands’ efficacy in LE pertains to their classification—L-type ligands (donors of two electrons, Lewis bases), X-type ligands (donors of one electron, anions), and Z-type ligands (acceptors of two electrons, Lewis acids). Maintaining charge neutrality, for instance, when substituting L-type ligands with X-type ligands, necessitates compensation for alterations in NP charge through a polar solvent [9].

The ligand coating of colloidal NPs directly affects their chemical compatibility and the ability to disperse them in water-based or oil-based suspensions. Notably, “water-soluble” nanoparticles offer significant advantages compared to their non-water-soluble counterparts in terms of environmental footprint [10] and offer significant advantages for important applications such as drug delivery [11,12].

Lead sulfide (PbS) is a common semiconductor belonging to the chalcogenide family (Groups IV–VI). It crystallizes in the rock salt crystal structure, characterized by a face-centered cubic lattice with Fm3m symmetry. At room temperature, the lattice constant of PbS is 5.963 Å, and its Bohr radius reaches 18 Å. As a semiconductor, PbS features a direct, narrow bandgap of 0.41 eV [13]. The synthesis of lead sulfide NPs can be achieved through a diverse array of methods. Several routes have previously been employed to create PbS NPs in organic and aqueous media, as well as within a polymer matrix [14]. The colloidal synthesis of PbS NPs is facilitated using single- or dual-source precursors, with a wide range of organic molecules employed as surfactants. Notable examples include octadecylamine (ODA) [13], oleylamine (OLA) [15,16], oleic acid (OA) [17], and polyvinylpyrrolidone (PVP) [18,19]. The choice of method and surfactant yields PbS NPs with distinct sizes and morphologies, such as cubes [13], rods [20], wires [21], stars [22], and various other shapes. This diversity in synthesis techniques and surfactants leads to a broad spectrum of PbS NP properties, particularly with respect to size and morphology. Consequently, the bandgap of PbS can be finely tuned over a substantial energy range, spanning from 0.6 eV [23] to as high as 2.1 eV [24]. PbS can exhibit either n-type or p-type semiconductivity, depending on the stoichiometry. Furthermore, experimental studies have demonstrated that nanostructured PbS shows remarkable optoelectronic properties for photonic applications, characterized by its non-linear optical activity, which exceeds that of GaAs by a factor of 30 and of CdSe nanoparticles by three orders of magnitude [25]. The inherent ability to finely adjust the bandgap of PbS NPs renders this material well suited for employment in photovoltaic devices [26]. Furthermore, the sizable Bohr radius of lead sulfide facilitates the realization of quantum confinement effects [27]. The wide-ranging tunability of PbS NPs’ bandgap renders them particularly appealing for deployment in mid- and near-infrared emission and detection applications [28]. Additionally, PbS finds applications in optoelectronic devices operating in mid- and near-infrared range [29]. Furthermore, PbS nanoparticles were successfully subjected to controlled aging, which enhanced their machinability properties, providing an advantage in industrial applications [30].

Previous studies were reported on the LE of PbS NPs, most notably replacing OA with water-soluble capping agents such as poly(acrylic acid) [31], 3-mercaptoproionic acid [32], and 2,3-dimercaptopropanesulfonate [33]. Additionally, LE has been performed from OA to carboxylic-acid-functionalized polyvinylpyrrolidone (PVP–COOH) on lead selenide (PbSe) NPs [34]. Johnson et al. successfully replaced OA with a PVP capping agent on β-NaYF^4+^/Er^3+^ NPs [35]. However, to the best of our knowledge, the procedure for replacing an ODA surfactant with PVP on PbS NPs has not been reported to date. Notable work in the synthesis of water-soluble PbS nanoparticles has been conducted by Leontidis et al. [36] and Bakueva et al. [28]. The capping agents used in these studies included poly(ethylene oxide), thiobutanol, thiohexanol, triglycolic acid, thioglycerol, and dithioglycerol. With regard to PVP-coated PbS NPs, several efforts have been directed towards the development of PbS–PVP nanocomposites [37,38,39,40,41]. Notably, in these studies, PVP was selected primarily for its polymeric properties rather than as a capping agent. Processes employing PVP as a surface capping agent have been particularly complex, frequently necessitating the use of hazardous hydrogen sulfide gas [42] or prolonged reaction times of up to 12 h [43,44]. More recently, Evstropiev et al. [18] and Bagrov et al. [19] successfully synthesized PVP-coated PbS NPs using lead nitrate and sodium sulfide as precursors.

This work aims to offer two alternative methods for the synthesis of PVP-coated PbS NPs. The first method involves the direct synthesis of the NPs with PVP serving as the surfactant, while the second method entails the synthesis of ODA-coated PbS NPs followed by an LE procedure from ODA to PVP, resulting in PVP-coated PbS NPs. Both methods have been shown to be eco-friendly compared to conventional synthesis techniques.

## 2. Materials and Methods

Lead (II) chloride (PbCl_2_, 99% trace metal basis), lead (II) perchlorate trihydrate (Pb(ClO_4_)_2_∙3H_2_O, ACS reagent 98%), thiourea (SC(NH_2_)_2_, ACS reagent ≥ 99.0%), potassium ethyl xanthogenate (also known as xanthate) (C_2_H_5_OCSSK, 96%), polyvinylpyrrolidone (PVP, (C_6_H_9_NO)_n_, average mol weight of 40,000), octadecylamine (ODA, C_18_H_37_NH_2_, ≥99.0% (GC)) *N*,*N*-dimethylformamide (DMF, HCON(CH_3_)_2_, 99.8% anhydrous) were all purchased from Sigma-Aldrich Israel Ltd. (Rehovot, Israel). Methanol (CH_3_OH), ethanol (C_2_H_5_OH), 2-propanol (ISO, (CH_3_)_2_COH_2_), diethyl ether (C_2_H_5_OC_2_H_5_), chloroform (CHCl_3_), *n*-hexane (CH_3_(CH_2_)_4_CH_3_), and ammonium hydroxide 25% (NH_4_OH) were obtained from Bio Lab. All materials were used without purification unless mentioned otherwise. Deionized water (DIW) was obtained from Millipore Direct Q3 with resistivity of 18.2 µΩ.

### 2.1. PVP-Coated PbS Nano-Cubes

The synthesis of PVP-coated PbS nano-cubes involved the following steps: Initially, 3.3 g of PbCl_2_ was dissolved in 10 mL of DIW within a 250 mL three-necked flask. An addition of 100 mL of ammonium hydroxide followed, and the flask underwent three cycles of vacuum and nitrogen gas purging. The solution was then heated to 60 °C under continuous stirring for 1 h. Four 50 mL test tubes, each containing 25 mL of methanol, were prepared to receive the solution. After centrifugation for 5 min at 4500 RPM, the liquid was decanted, and the slurry was filled with methanol, followed by centrifugation under the same conditions. The resultant material was dried under a vacuum. The molar concentration of the lead chloride was 0.1 M. Subsequent X-ray diffraction (XRD) analysis confirmed a satisfactory match to Pb_4_O_3_Cl_2_∙H_2_O, and this product was employed as the lead precursor. For the synthesis of PbS nano-cubes, 50 mg of the lead precursor was dissolved in 15 mL of DIW within a 50 mL three-necked flask. Separately, 800 mg of PVP was fully dissolved in 6 mL of DIW and subsequently added to the flask. The solution was then heated to 100 °C while maintaining constant stirring and N_2_ purging. After 10 min, 47.7 mg of thiourea, dissolved in 5 mL of DIW, was injected into the flask. The solution gradually transformed from white to dark brown following thiourea injection, signifying the formation of PbS NPs. The solution was kept at 100 °C for an additional 2 h and was subsequently transferred into a 50 mL test tube containing 25 mL of methanol, followed by centrifugation for 5 min at 4500 RPM. The liquid was removed, and the test tube was subjected to a mixture of methanol and DIW (1:1) before undergoing another round of centrifugation. The resulting slurry was dried and maintained under a vacuum. The final molar concentrations were 2 mM, 0.7 mM, and 24.0 mM for Pb_4_O_3_Cl_2_, PVP, and thiourea, respectively.

### 2.2. Ligand Exchange from ODA to PVP in PbS Truncated Nano-Cubes

#### 2.2.1. Synthesis of ODA Coated PbS Truncated Nano-Cubes

The procedure for synthesizing ODA-coated PbS truncated nano-cubes was adopted from Rabkin et al. [13]. In this process, 3000 mg of potassium ethyl xanthogenate was dissolved in 400 mL of DIW within a 500 mL beaker. Separately, 4305 mg of lead perchlorate trihydrate was dissolved in 100 mL of DIW within a 200 mL beaker. The final molar concentrations were 0.037 M and 0.018 M for potassium ethyl xanthogenate and lead perchlorate trihydrate, respectively. The resulting solutions were combined to produce a very light brown mixture. This mixture was then divided into 10 separate 50 mL test tubes, which were subjected to centrifugation for 5 min at 6000 RPM. The liquid was decanted, and the residual slurry was washed with DIW before undergoing centrifugation for 5 min at 3000 RPM. This washing procedure was repeated four additional times. Following washing, the sediment was dried and stored under a vacuum. This protocol yielded lead-ethyl xanthate—a single-source precursor (SSP) utilized for PbS NP synthesis. To initiate the synthesis of PbS NPs, a silicon oil bath was heated to 95 °C. A glass test tube containing 1530 mg of ODA was heated in the silicon oil bath until the ODA was completely melted. Subsequently, 117 mg of lead-ethyl xanthate was introduced to the glass tube while the system was kept under continuous stirring and nitrogen purging. After 5 min, the synthesis process was halted by adding methanol. The resulting mixture was then transferred into a 50 mL test tube, which was subsequently centrifuged for 5 min at 4500 RPM. Excess solvent was removed, and the slurry was subjected to a total of 3 washes using a solvent mixture of chloroform and methanol in a 1:2.5 ratio. The molar concentrations of the SSP and the surfactant were 3.0 M and 0.12 M, respectively.

#### 2.2.2. Ligand Exchange from ODA to PVP

The process of ligand exchange, transitioning from ODA to PVP, was adapted and scaled up from the method outlined by Johnson et al. This prior study focused on ligand exchange from oleic acid to PVP on β-NaYF_4_:Yb^3+^/Er^3+^ NPs [35]. In the adapted procedure, 1.75 mg of ODA-coated PbS powder was dispersed in 0.4 mL of chloroform within a 50 mL three-necked flask. To this flask, 5 mL each of chloroform and DMF, as well as 100 mg of PVP, were introduced. The flask underwent three repetitions of vacuum and nitrogen gas cycles. The mixture was then gently heated to attain a reflux state for a duration of 6 h. Throughout the procedure, the temperature was carefully controlled to maintain the gentle reflux, fluctuating between 88 °C and 114 °C. Following synthesis, the solution was gradually poured onto 25 mL of diethyl ether within a 50 mL test tube, subsequently undergoing centrifugation for 5 min at 4500 RPM. After decanting the solvent, the resulting slurry underwent a washing process involving 35 mL of diethyl ether and 15 mL of methanol. Centrifugation at 3000 RPM for 5 min followed, culminating in drying and storage under vacuum. The molar concentrations of the PbS NPs and the PVP amounted to 0.7 mM and 0.24 mM, respectively.

### 2.3. Characterization Methods

**X-ray diffraction**

Structural evaluation of the NPs was carried out with a Panalytical Empyrean powder diffractometer (Malvern Panalytical B.V., Almelo, The Netherlands) equipped with a PIXcel linear detector. Data were collected in the 2θ/θ geometry with a scan run of 8 min and steps of ~0.033° using Cu Kα radiation (λ = 1.5405 Å). The 2θ scan range was 10–60°.

**Transmission electron microscopy**

Transmission electron microscopy (TEM) and selected area electron diffraction (SAED) were carried out using an FEI Tecnai T12 operating at 120 kV. High-resolution transmission electron microscopy (HRTEM) imaging was carried out using a JEOL JEM-2100F analytical TEM (purchased from JEOL UK Ltd., Welwyn Garden City, United Kingdom) operating at 200 keV equipped with a GATAN 894 US1000 camera. Energy-dispersive X-ray spectroscopy (EDS) was performed at 200 keV using a JEOL JEM-2100F TEM using an Oxford Instruments X-Max 65T SDD detector with ±2% accuracy for heavy elements such as lead (Pb) and tin (Sn) and ±5% for the lighter elements such as sulfur (S) and oxygen (O). Scanning TEM (STEM) images were taken using a GATAN 806 HAADF STEM detector (Gatan Inc., Pleasanton, CA, USA). The probe size during the analysis was set to 1 nm. INCA software (v. 5.05) and Aztec software (v. 3.3, Abingdon, Oxfordshire, UK) were used for the EDS data analysis. Quantitative analysis was performed by the standardless Cliff−Lorimer method.

**Fourier-transformed infrared spectroscopy**

Fourier-transform infrared (FTIR) spectroscopy was carried out using a Nicolet 8700 FTIR spectrometer (Thermo Electron Corporation, Madison, WI, USA) fitted with a DTGS detector with a Diamond ATR accessory. All single-beam spectra were measured against a clean crystal background. The spectra were recorded in the range from 4000 to 700 cm^−1^, with 36 scans with an optical velocity of 0.47 and an aperture of 100. The FTIR data were collected using OMNIC software (version 9). All spectra were present after atmospheric suppression and background correction.

**Thermal gravimetric analysis**

Thermal gravimetric analysis (TGA) was carried out using a TA instrument TGA 500 (New Castle, DE, USA). The measurements were conducted in a N_2_ atmosphere with a heating rate of 10 °C per min from room temperature to 1000 °C.

## 3. Results and Discussion

### 3.1. PVP-Coated PbS Nano-Cubes

**Figure 1:**
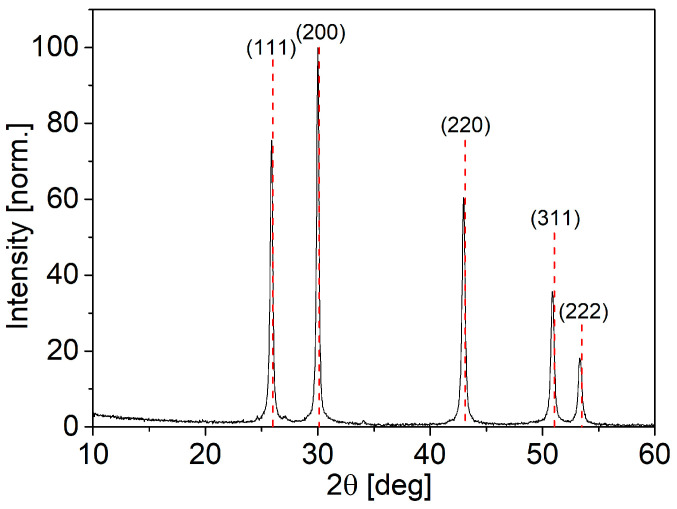
XRD diffractogram of PbS NPs. The dashed red lines represent the peak positions of rocksalt PbS.

The crystallographic properties of the NPs were evaluated using XRD analysis, as shown in Figure 1. The diffractogram of the NPs is compared to the diffractogram of rock salt PbS (Fm$\bar{3}$m), which is shown with red dotted vertical lines, indicating that the NP sample has an identical crystal structure to the reference, with no crystalline foreign phases present in the sample. The coherence length of the PbS NPs was determined using the Scherrer equation by performing Gauss fitting at 2θ = 29.99° on the XRD pattern, and an FWHM value of 0.27662° was obtained. When converted to radians, this value becomes β = 4.8 × 10^−3^ rad. Using a shape factor K of 0.9 and an X-ray wavelength of 0.15405 nm, the Scherrer equation yielded a coherence length of ~30 nm for the PbS NPs.

**Figure 2:**
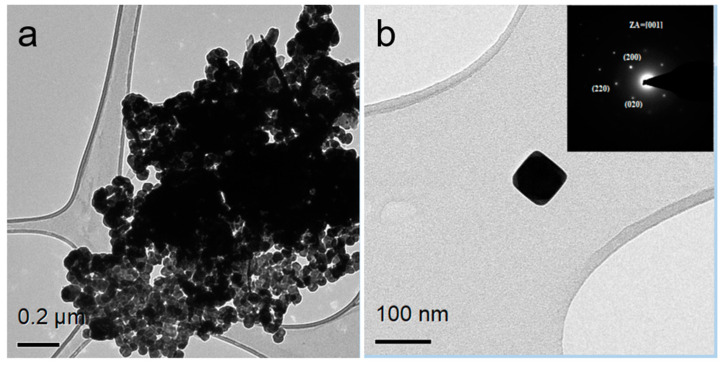
TEM images of PbS nano-cubes. (**a**) An aggregate of nano-cube-shaped NP with different orientations, (**b**) a magnified image of a nano-cube of PbS with a size of ~80 nm in the insert—SAED shows rocksalt PbS where the ZA = [001].

The morphological characterization of the PbS NPs was conducted using TEM. The NPs were dispersed in ethanol and deposited onto holey carbon film on a copper TEM grid. Figure 2a presents the characteristic morphology of the PbS NPs, illustrating their cube-like shape, as shown in the high magnification image in Figure 2b. Additionally, SAED was performed, generating a diffraction pattern corresponding to the rocksalt structure of PbS with a [001] zone axis (ZA). A semi-quantitative STEM-EDS elemental analysis for a typical nanocrystal directly synthesized in the presence of PVP is provided in Figure S1 and Table S1 (Supplementary Information).

**Figure 3:**
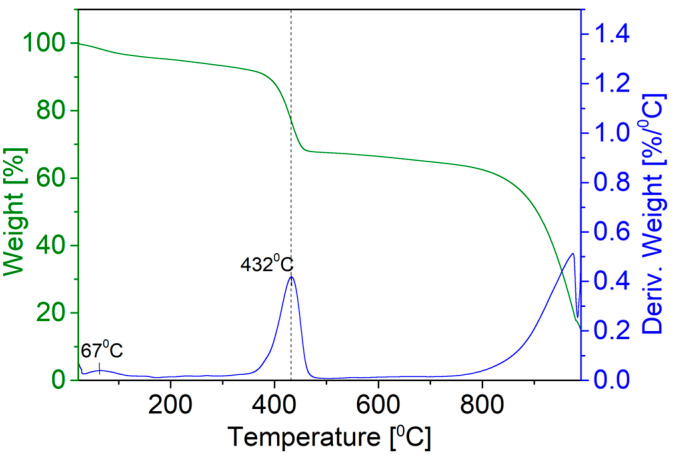
TGA of PVP-coated PbS NP powder. The green curve represents the weight percentage of the PbS NP powder as a function of temperature. The blue curve represents the derivative of the weight percentage.

The surfactant coverage on the PbS NPs was assessed using TGA. A total of 14.6 mg of dry PbS nanoparticle powder was subjected to heating, with the temperature ranging from room temperature up to 1000 °C. The TGA results are presented in Figure 3. The initial weight loss observed in the temperature range of approximately 31 °C to 200 °C, corresponding to around 4% of the total weight, can be attributed to the evaporation of organic solvent, water molecules, and other low-boiling-point impurities in the sample. The subsequent weight loss occurring from approximately 200 °C to 601 °C, accounting for about 26% of the total weight, likely corresponds to the decomposition of the PVP molecules that coat the PbS NPs. The derivative weight curve exhibits a peak at around 432 °C, in alignment with the chemical destruction temperature of the PVP coating on the surface of NPs [45]. From this analysis, ~70% out of the 14.6 mg of powder used in the analysis is PbS.

### 3.2. Ligand Exchange from ODA to PVP in PbS Truncated Nano-Cubes

**Figure 4:**
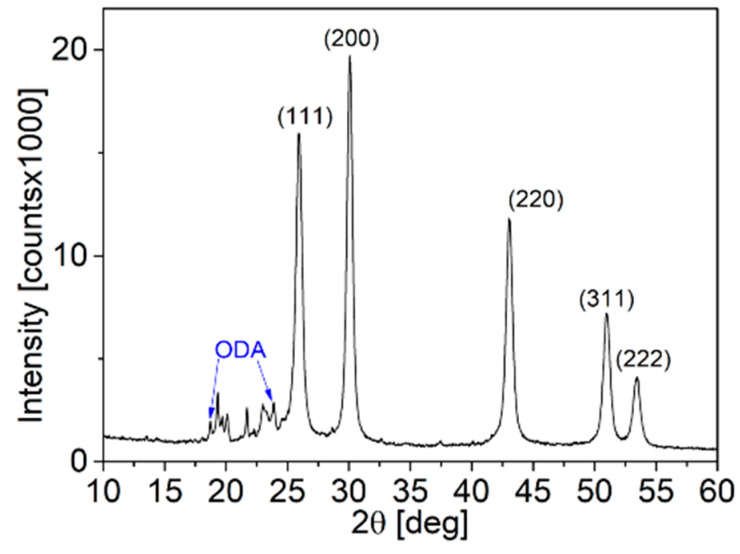
XRD diffractogram of ODA-coated PbS NPs.

The synthesis procedure employed was previously demonstrated by Rabkin et al. to yield PbS truncated nano-cubes [13]. As depicted in Figure 4, the diffractogram of the NP powder corresponds to the rocksalt phase of PbS. Bragg peaks appearing at 2θ < 26.00° are attributed to excess crystalline ODA. The coherence length of the NPs was determined using the Scherrer equation by conducting Gaussian fitting to the diffractogram at 2θ = 30.07°, and an FWHM value of 0.55017 degrees (converted to radians as β = 9.6 × 10^−3^) was obtained. Substituting this value into the Scherrer equation indicates that the coherence length of the PbS NPs is approximately 15 nm.

**Figure 5:**
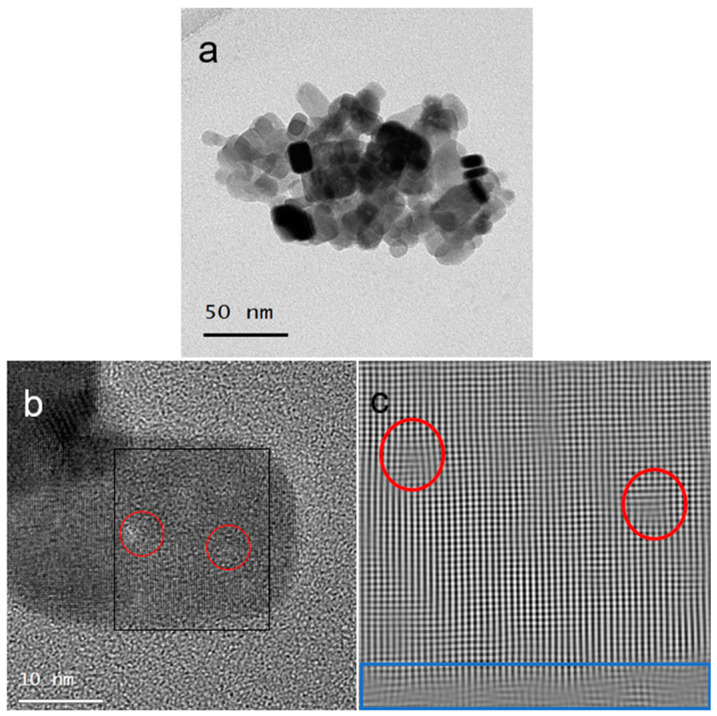
Micrographs of PbS truncated nano-cubes. (**a**) A TEM image of a PbS aggregate. (**b**) An HRTEM image of an individual PbS nanoparticle. (**c**) An IFFT of the particle in (**b**). Red circles in (**b**,**c**) denote regions with structural defects in the NPs.

The morphology of the PbS NPs was assessed using HRTEM. The PbS powder was dispersed in *n*-hexane, and holey carbon grids were immersed in the dispersion and subsequently allowed to dry. Micrographs in Figure 5a,b distinctly reveal the prevalent truncated nano-cube morphology of the NPs. Some NPs exhibit brighter circular regions, as evident in Figure 5b. These brighter circles likely indicate structural defects in the NPs. Additional insights into the defects are discernible from the inverse fast Fourier transform (IFFT) image, as depicted in Figure 5c. This IFFT was acquired from the particle shown in Figure 5b, delineated by a black rectangle. Notably, dislocation and corresponding features are highlighted by red circles in the IFFT image. The apparent amorphous segment at the bottom of the IFFT image (marked by the blue rectangle) is likely to result from the surfactant layer on the particle surface.

**Figure 6:**
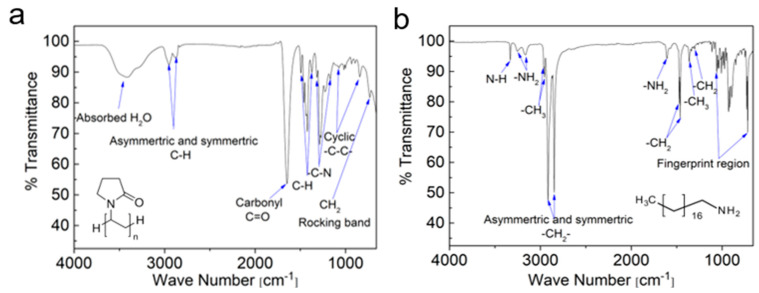
FTIR spectra and molecular structure of (**a**) pure PVP and (**b**) pure ODA.

Both ODA and PVP have been extensively researched and utilized within the realm of colloidal synthesis of NPs. ODA is characterized as a hydrophobic molecule featuring an aliphatic chain comprising 18 carbon atoms ending with an amine headgroup. On the other hand, PVP is classified as a water-soluble polymer derived from *N*-vinylpyrrolidone monomers. Each monomer within the PVP encompasses a pair of carbon atoms, with one of them being bonded to a lactam group, which is essentially a cyclic amide. The molecular structures of these two ligands are visually represented in Figure 6. Notably, PVP40, which references PVP with an average chain comprising approximately 360 monomers, is notably more substantial in size compared to ODA. This size disparity may potentially lead to compromised conductivity and photoluminescence of NPs when coated with PVP, especially when compared with the relatively shorter chains of ODA [24,46]. However, the application of PVP ligands as surfactants in NP synthesis can result in hydrophilic NPs, serving as an advantageous attribute. Due to the inherent functional groups in ODA and PVP, an analytical technique, such as FTIR spectroscopy, can be employed to discern the presence of these functional groups on the surface of the NP powders. The absorbance peaks corresponding to these functional groups are detailed in Table 1 [37,47], which is accompanied by the assignment of the peaks within the FTIR spectra, as depicted in Figure 6. This analytical approach can effectively serve as a means of gauging the efficacy of the LE procedure.

**Figure 7:**
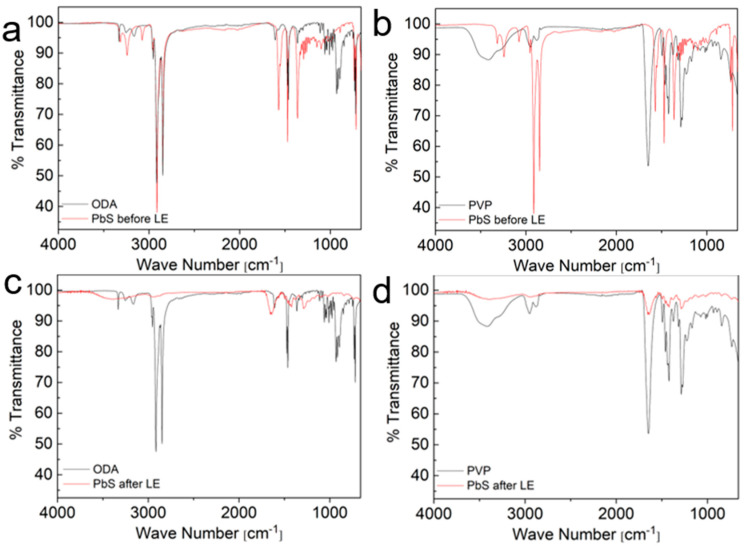
FTIR measurements of PbS NPs: (**a**) PbS before LE compared to ODA; (**b**) PbS before LE compared to PVP; (**c**) PbS after LE compared to ODA; and (**d**) PbS after LE compared to PVP.

Figure 7 presents the FTIR spectra of the PbS NPs before and after the LE process, alongside the spectra of the two pure ligands, ODA and PVP, for comparison. The FTIR spectra of the as-synthesized PbS NPs prior to the LE procedure exhibit a pronounced resemblance to the FTIR spectra of pure ODA. This observation provides further support for the effective coverage of the PbS NPs by ODA ligands. Notably, a discernible red shift is evident in the amine-related peaks. Specifically, the asymmetric and symmetric –N–H stretching peaks exhibit a shift from 3255 cm^−1^ to 3241 cm^−1^ and from 3163 cm^−1^ to 3076 cm^−1^, respectively. Furthermore, the –NH_2_ stretching peak undergoes a shift from 1607 cm^−1^ to 1569 cm^−1^. This alteration in the amine peaks implies that the bonding of ODA ligands to the NP surface predominantly occurs via the amine functional group. Interestingly, several characteristic peaks in the 750–1080 cm^−1^ region, attributed to different modes of the –C–C– bonds in ODA, are absent from the PbS spectra. This absence of peaks in that region suggests a robust attachment of the ODA ligands to the NP surface. In contrast, greater variances exist between the FTIR spectra of the PbS NPs and pure PVP, implying differences in the bonding interactions and chemical environments associated with the pure and bound ligands.

**Figure 8:**
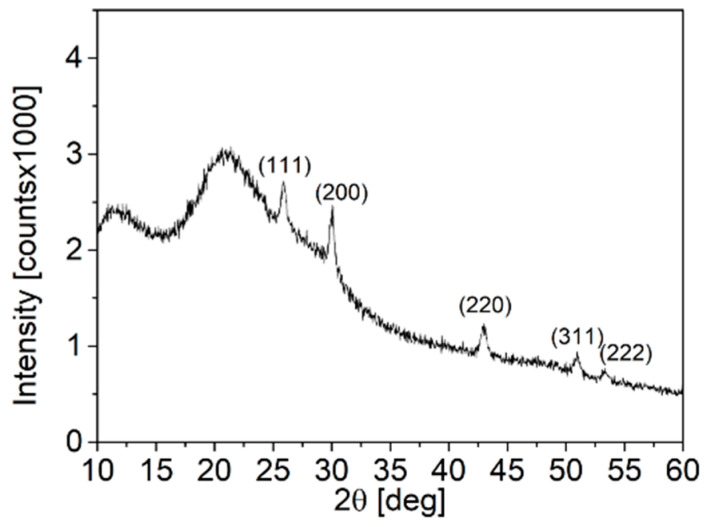
XRD of PbS NPs following ligand exchange.

Upon performing the LE procedure, all the characteristic peaks associated with ODA completely disappear from the PbS FTIR spectra, and the absorbance peaks align closely with those of pure PVP. This indicates a successful replacement of ODA ligands with PVP ligands on the PbS NP surface. However, unlike the red shifts observed in the amine-related peaks during the pre-LE analysis, no shifts are observed in the PVP functional group peaks in the PbS spectra. There are a couple of possible explanations for this lack of shift in the PVP-related peaks. Firstly, it could be due to weaker bonding interactions between PVP and the NP surface following LE, such that the binding is not strong enough to cause significant peak shifts in the FTIR spectra. Alternatively, there might be an excess of unbound PVP in the powder, resulting in a smaller fraction of PVP molecules that are bonded to the NP surface. This possibility is supported by the XRD analysis shown in Figure 8. While the characteristic Bragg peaks of rocksalt PbS indicate that the NP structure remains unchanged after the LE procedure, there is an amorphous region in the diffractogram, likely attributed to a significant presence of organic substances such as PVP. By comparing the XRD diffractogram of PVP-coated PbS NPs after LE with the diffractogram of PbS NPs synthesized directly with PVP as the surfactant in Figure 1, it can be inferred that there is an excess of PVP molecules in the post-LE powder. This reinforces the notion that the lack of peak shifts in the PVP-related FTIR peaks could be due to the significant presence of unbound PVP molecules in the powder.

**Figure 9:**
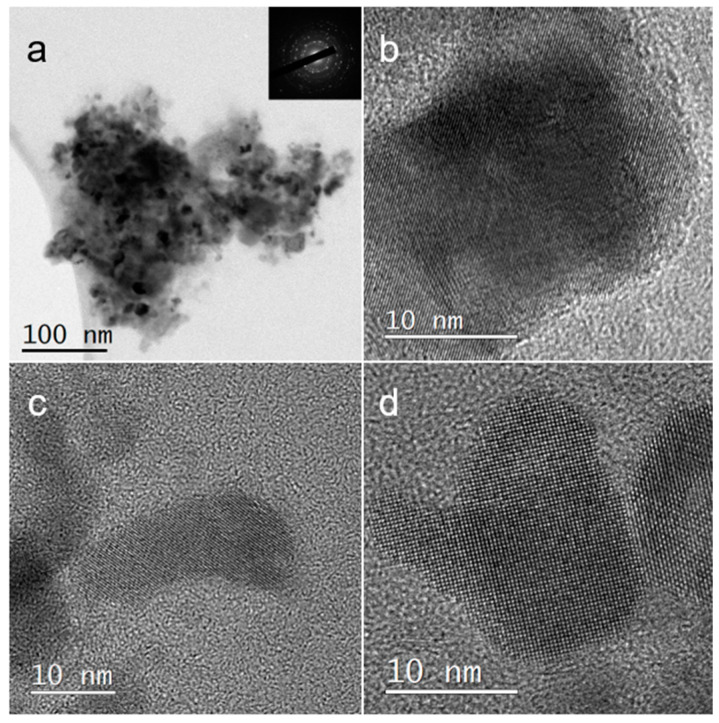
TEM images of PbS NPs after LE. (**a**) Bright-field image of PbS nano-cubes. (**b**) HRTEM of PbS nano-cubes. (**c**,**d**) HRTEM of oddly shaped PbS NPs. Inset in (**a**) shows an electron diffraction pattern obtained from the NP aggregate shown in (**a**).

Maintaining the original morphology of the NPs is crucial for a successful ligand exchange procedure, as changes in morphology could impact the properties and applications of the NPs. Comparing the typical morphology of the ODA-coated NPs in Figure 6 to the morphology of the PVP-coated NPs shown in Figure 9a,b indicates that the majority of NPs retained their original cube-like morphology and size even after the ligand exchange procedure. However, Figure 9c,d show cases where NPs with different morphologies are present. The EDS measurements presented in Table 2 confirm that these oddly shaped NPs are indeed PbS NPs. Determining whether these oddly shaped NPs were formed during the initial synthesis or were a result of the LE procedure is challenging and requires further investigation. These data suggest that while most of the NPs maintain their original morphology, there are some NPs that show alterations or variations in the morphology, possibly due to the ligand exchange process or inherent variability in the initial NP synthesis. Further characterization and analysis will help elucidate the origin of these differently shaped NPs in the future.

## 4. Conclusions

This research has contributed two previously unreported approaches to synthesizing PVP-coated PbS nano-cubes, employing ligand exchange and the direct synthesis of PbS nano-cubes. As previously mentioned, extensive research has been conducted on the colloidal synthesis of PbS. This also holds true for the utilization of PVP as a surfactant in colloidal synthesis. Extensive efforts have been dedicated to the development of PbS–PVP nanocomposites [37,38,39,40,41]. It is noteworthy that PVP was primarily chosen for its polymeric properties rather than its capping agent functionality. Synthesis processes employing PVP molecules as surfactants have been notably intricate, often involving the use of hazardous H_2_S gas [42] or extended reaction durations [43,44] of up to 12 h. In contrast, the synthesis technique detailed in this work is significantly more efficient, requiring only 2 h, and is conducted in a N_2_ environment. To the best of our knowledge, the utilization of Pb_4_O_3_Cl_2_ as a source for lead cations in PbS NP synthesis has not been previously documented. This lead oxide chloride phase was reported to be a residue of various lead manufacturing techniques [48,49]. The capacity to utilize this phase as a precursor for an alternative process, rather than discarding it as waste, contributes to a more environmentally friendly approach in the manufacturing of lead. The ligand exchange process entailed the well-established and facile procedure of synthesizing ODA-coated PbS truncated nano-cubes, followed by the replacement of ODA surfactant with PVP surfactant on the surface of PbS NPs post-synthesis. Analyses using FTIR spectroscopy and TGA confirmed the complete replacement of ODA surfactant with PVP. Furthermore, TEM, STEM, and EDS elucidated that the majority of the nanoparticles maintained their original morphology after the ligand exchange procedure. This ligand exchange procedure is likely to be applicable to other ODA-coated metal chalcogenide nanoparticles, such as ZnS, SnS, and CdS. The second method involving the direct synthesis of PVP-coated PbS nanoparticles in the aqueous phase yielded cubic morphology with typical dimensions of approximately 30 nm. This synthesis procedure proved to be facile and relatively environmentally friendly, as it did not require the use of toxic materials other than the lead precursor itself.

## Figures and Tables

**Table 1:** IR absorbance bands for the different functional groups present in ODA and PVP.

ODA		PVP	
Absorbance Band cm^−1^	Functional Group	Absorbance Band cm^−1^	Functional Group
3330	N–H crystalline	3400	Absorbed H_2_O
3255	Asymmetric and symmetric –NH_2_ stretching mode	2950	Asymmetric and symmetric C–H stretching mode
3163		2873	
2962	Asymmetric and symmetric methyl –CH_3_ stretching mode	1647	Carbonyl C=O stretching mode
2953			
2915	Asymmetric and symmetric –CH_2_– stretching	1493	In plane C–H bending of different –CH_2_ and C–H
		1460	
2847		1435	
		1421	
		1373	
1607	–NH_2_	1316	–C–N stretching mode
		1286	
1471	–CH_2_ splitting	1226	–C–N stretching mode
1461		1170	
1364	–CH_3_ “umbrella”	1074	Cyclic –C–C– stretching mode
		1017	
1338		933	
		844	
1320	Twisting–rocking and wagging –CH_2_–	731	Amide or CH_2_ rocking band
1303			
719–1060	Fingerprint region		

**Table 2:** Semi-quantitative STEM-EDS analysis of PbS NPs after LE.

Spectrum Label	Figure 7b at%	Figure 7c at%	Figure 7d at%
S	53	52	50
Pb	47	48	50
Total	100	100	100

## Data Availability

The data presented in this study are available on request from the corresponding author. The raw data are available on the repository of the Ilse Katz Institute for Nanoscale Science and Technology at BGU.

## Supplementary Materials

The following supporting information can be downloaded at: https://www.mdpi.com/article/10.3390/nano14141235/s1, Figure S1: Typical STEM image of a PbS nano-cube synthesized directly in presence of PVP.; Table S1: Semi-quantitative EDS/STEM analysis from the particle shown in Figure S1.
