# Supplementary material for: Water-Soluble Lead Sulfide Nanoparticles: Direct Synthesis and Ligand Exchange Routes
Source: Nanomaterials (Basel). 2024 Jul 22;14(14):1235. doi: 10.3390/nano14141235 (PMC11280317; doi:10.3390/nano14141235)
Supplement: Supplementary file 1 [file nanomaterials-14-01235-s001.zip › nanomaterials-3095481-supplementary.pdf]

nanomaterials

Supplementary materials

Water-Soluble Lead Sulfide Nanoparticles: Direct Synthesis and Ligand Exchange Routes

Saar Pfeffer [1,2], Vladimir Ezersky [2], Sofiya Kolusheva [2] and Yuval Golan [1,2,*]

[1] Department of Materials Engineering, Ben-Gurion University of the Negev, Beer-Sheva 8410501, Israel; saarpf@post.bgu.ac.il
[2] Ilse Katz Institute for Nanoscale Science and Technology, Ben-Gurion University of the Negev, Beer-Sheva 8410501, Israel; ezersky@bgu.ac.il (V.E.); kolushev@bgu.ac.il (S.K.)
* Correspondence: ygolan@bgu.ac.il

S.1. EDS analysis

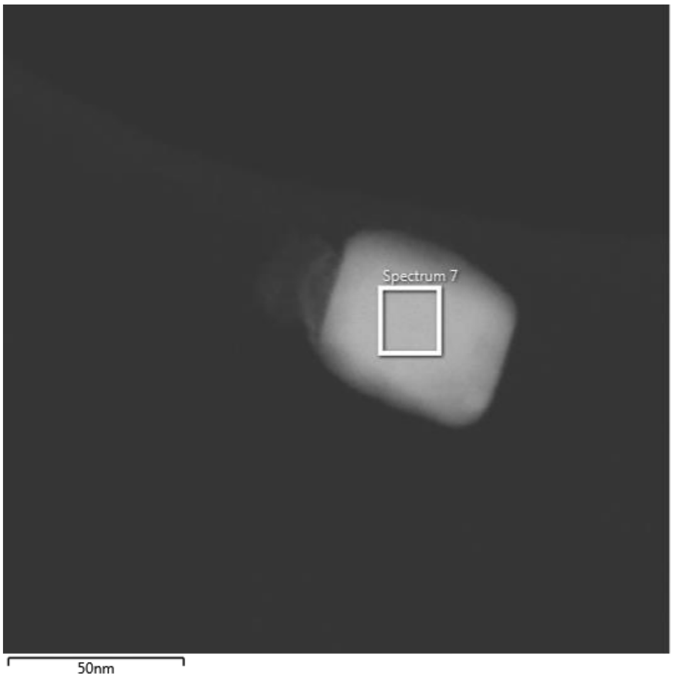

Figure S1. Typical STEM image of a PbS nano-cube synthesized directly in presence of PVP.

Table S1. Semi-quantitative EDS/STEM analysis from the particle shown in Figure S1.

Spectrum Label	Spectrum 7 (atomic %)
S	49
Pb	51
Total	100
